# PRKN regulates inner mitochondrial membrane PHB2 during mitophagy

**DOI:** 10.1080/27694127.2022.2164643

**Published:** 2023-01-16

**Authors:** Shan Sun, Hongfeng Wang, Qilian Ma, Ningning Li, Mian Cao, Kin Yip Tam, Zheng Ying

**Affiliations:** a Jiangsu Key Laboratory of Neuropsychiatric Diseases and College of Pharmaceutical Sciences, Soochow University, Suzhou, Jiangsu 215123, China; b Faculty of Health Sciences, University of Macau, Taipa, Macau, China; c Programme in Neuroscience and Behavioural Disorders, Duke-NUS Medical School, 169857, Singapore

**Keywords:** MAP1LC3B/LC3B, mitophagy, PHB2, PRKN, ubiquitination

## Abstract

PINK1 (PTEN induced kinase 1) and PRKN-mediated mitophagy is an important mitochondrial quality control pathway which selectively degrades damaged mitochondria and is tightly associated with neurodegenerative diseases, including Parkinson disease and amyotrophic lateral sclerosis. The current model of PINK1-PRKN-mediated mitophagy is that PRKN ubiquitinates multiple outer mitochondrial membrane (OMM) proteins, and then the ubiquitin chains on the OMM interact with autophagy receptors which bind Atg8-family protein labeled phagophores. However, little work has been focused on the PRKN-mediated ubiquitination of inner mitochondrial membrane (IMM) proteins during mitophagy. Our recent work revealed that PRKN binds and ubiquitinates PHB2 (prohibitin 2), an essential IMM protein which was previously recognized as a mitophagy receptor, after the OMM is ruptured by proteasomal degradation. Using biochemical and microscopy approaches, we found that mutations of PRKN-targeted ubiquitination sites on PHB2 decrease the recognition of damaged mitochondria by the phagophore and the clearance of damaged mitochondria. In conclusion, our findings revealed a critical role for PRKN-PHB2 interaction in mitochondrial quality control by regulating IMM-associated recognition of mitochondria by the autophagy machinery.

Parkinson disease (PD), one of the most common neurodegenerative disorders, affects 1-2% of the world population over age 60 and has been considered as a mitochondrial disease of aging. Accumulating evidence has linked two players, PINK1/PARK6 and PRKN/Parkin/PARK2, encoded by two recessive PD genes, in a common pathway regulating the quality control of mitochondria. In 1998, Shimizu identified *PRKN* as a PD-linked gene and more than 100 pathogenic PRKN loss-of-function mutations have been found after that. Among many of the PD-associated proteins, PRKN is very interesting because it is an E3 ubiquitin (UB) ligase that can regulate mono- or poly-ubiquitination of many interacting substrates, including PD-linked PARK7/DJ-1, protein phosphatase PTPN5/STEP61 and the transcriptional regulator ZNF746/PARIS that are relevant to PD pathogenesis, synaptic proteins (DLG4/PSD-95 and SNCAIP/Synphilin-1), regulator in immune system (NLRP3), and a series of outer mitochondrial membrane (OMM) proteins (RHOT/Miro, VDAC1 and MFN1), thereby linking many biological components to the pathological mechanism underlying PD.

Among many hypotheses of PD pathogenesis, dysfunctional mitophagy has received considerable attention. Mitophagy is a major pathway for selective removal of damaged mitochondria in cells, which is a vital process for cell homeostasis and is tightly linked to neurodegenerative disorders. Mitophagy begins from the stabilization of PINK1 which phosphorylates nearby UB. PRKN interacts with the phosphorylated UB (p-UB), and then is phosphorylated and activated by PINK1. Once activated, PRKN links p-UB to OMM proteins, thereby triggering OPTN and CALCOCO2/NDP52 translocation to ubiquitinated OMM proteins via UBAN domains in OPTN and CALCOCO2. After that, OPTN and CALCOCO2 bind Atg8-family proteins (including MAP1LC3/LC3 and GABARAP subfamilies), embedded in the phagophore, via LC3-interacting region/LIR domains, resulting in the degradation of damaged mitochondria by the fusion of mitophagosomes (autophagosomes selectively containing mitochondria) and acidic lysosomes (Figure 1).

**Figure 1.**
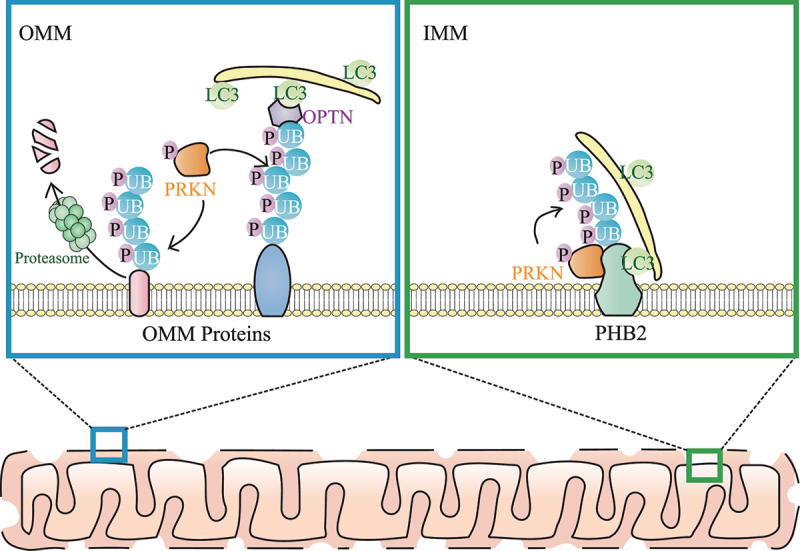
Model for PRKN-mediated ubiquitination on the OMM and IMM during mitophagy. Upon mitochondrial damage, both ubiquitin (UB) and PRKN are phosphorylated by PINK1. OMM proteins are ubiquitinated by PRKN and many ubiquitinated OMM proteins are degraded by proteasomes, leading to OMM rupture. In addition, autophagy receptors (including OPTN) link the ubiquitinated OMM and phagophore (which contains LC3s including LC3B) together. After OMM rupture, the IMM autophagy receptor PHB2 is also ubiquitinated by PRKN, and PRKN-mediated ubiquitination of PHB2 enhances PHB2-LC3 interaction, thereby promoting the phagophore recognition of the “entire” mitochondria during mitophagy.

The ubiquitination amplifier system and positive feedback loop on OMM are critical and multifarious for PINK1-PRKN-mediated mitophagy. In particular, PRKN amplifies the UB-signal in mitophagy by accumulating additional UB chains onto the OMM, where UB is phosphorylated by PINK1. Subsequently, the additional recruitment of UB further facilitates nearby PRKN activation in a PINK1-dependent positive feedback loop. However, it is unclear whether an inner mitochondrial membrane (IMM) mitophagy amplifier system exists. Our recent work provides evidence that PHB2 (prohibitin 2), a critical IMM mitophagy receptor which directly interacts with LC3B and thereby facilitates phagophore recognition of mitochondria, interacts with the RING1 domain of PRKN and is ubiquitinated by PRKN on K142 and K200 sites after OMM rupture [1]. Surprisingly, PRKN assembles K11- and K33-linked p-UB chains on PHB2, but not K6-, K48- or K63-linked p-UB chains that are commonly found on the OMM during mitophagy. Moreover, biochemical and microscopy experiments identify K11- and K33-linked ubiquitination of PHB2 acts as an “amplifier signal”, but not the “degradative signal” of PHB2, when playing its role as the mitophagy receptor on IMM. Importantly, we find that the clearance of damaged mitochondria is relatively slower if we knock down *PHB2* or abolish PRKN-mediated ubiquitination on PHB2. Therefore, like the classic amplifier role of PRKN on the OMM, we find that the PRKN-mediated amplifier role may also exist on the IMM, supported by the evidence that PRKN recruits additional UB chains to amplify the PHB2-LC3B signal on the IMM.

Given the fact that the IMM is ubiquitinated during mitophagy, there is a possibility that other autophagy receptors such as OPTN and CALCOCO2 can be recruited to the ubiquitinated IMM through UB-UBAN interaction and further promote the recruitment of the autophagosomal initiator ULK1 complex, thereby generating more LC3B on the IMM. In addition, PHB2 may not only directly interact with LC3B via LC3-interacting regions, but may also indirectly interact with LC3B via UB-associated OPTN and CALCOCO2. These possibilities support the idea that PRKN-mediated ubiquitination can amplify the translocation of LC3B on the IMM and can result in the rapid covering of the IMM with a phagophore.

Taken together, our recent study supports a novel mechanistic model for mitophagy that ubiquitination on both the OMM and IMM contribute to efficient mitophagy clearance of damaged mitochondria (Figure 1). OMM ubiquitination is necessary for OMM rupture, and IMM ubiquitination is important for efficient phagophore recognition of the entire mitochondria. As an IMM protein, PHB2 not only plays a role as a mitophagy receptor, but also acts as a molecular target for PRKN-mediated IMM ubiquitination, thereby triggering the amplification of LC3B recruitment.
